# Endocrine disruption rewards: bisphenol-A-induced reproductive toxicity and the precision ameliorative potential of flavonoids in preclinical studies. A systematic review and meta-analysis

**DOI:** 10.3389/ftox.2025.1687862

**Published:** 2025-11-24

**Authors:** Michael Ben Okon, Ilemobayo Victor Fasogbon, Dominic Swase, Reuben Samson Dangana, Wusa Makena, Vivian Onyinye Ojiakor, Ekom Monday Etukudo, Joan Chebet, Angela Mumbua Musyoka, Sandra Etumah Ifie, Herbert Mbyemeire, Solomon Adomi Mbina, Okechukwu Paul-Chima Ugwu, Augustine Oviosun, Ibe Micheal Usman, Josiah Eseoghene Ifie, Loganathan Rangasamy, Olubukola Sinbad Olorunnisola, Philippe Mounmbegna, Sana Noreen, Patrick Maduabuchi Aja

**Affiliations:** 1 Department of Biochemistry, Faculty of Biomedical Sciences, Kampala International University, Western Campus, Bushenyi, Uganda; 2 Discipline of Genetics, School of Life Sciences, University of KwaZulu-Natal, Westville Campus, Durban, South Africa; 3 Department of Human Anatomy, Kampala International University, Bushenyi, Uganda; 4 Department of Publication and Extension, Kampala International University, Bushenyi, Uganda; 5 Department of Science, Valley University of Science and Technology, Bushenyi, Uganda; 6 Centre for Biomaterials, Cellular and Molecular Theranostics (CBCMT), Vellore Institute of Technology, Vellore, India; 7 University Institute of Diet and Nutritional Sciences, The University of Lahore, Lahore, Pakistan

**Keywords:** bisphenol A, flavonoids, endocrine disruption, oxidative stress, reproductive toxicity, preclinical studies, meta-analysis

## Abstract

**Introduction:**

Bisphenol A (BPA), a pervasive endocrine-disrupting chemical, impairs male reproductive health via oxidative stress, hormonal dysregulation, and hypothalamic–pituitary–gonadal (HPG) axis disruption. Flavonoids, widely present in plant-derived foods and medicinal herbs, possess antioxidant and steroidogenic modulatory properties that may counteract BPA toxicity, yet preclinical findings remain inconsistent. This study aims to systematically evaluate and quantitatively synthesize preclinical evidence on the protective effects of flavonoids against BPA-induced male reproductive toxicity.

**Methods:**

Using PRISMA 2020 guidelines, Web of Science, Scopus, and PubMed were searched up to September 2024. Eligible studies involved BPA exposure in male rodents with flavonoid co-treatment and reported reproductive endpoints. Hormonal and oxidative stress biomarkers were pooled using a random-effects model, expressed as standardized mean differences (SMDs), with heterogeneity assessed by I^2^ statistics. Twenty studies were included.

**Results:**

BPA significantly reduced testosterone (SMD = −4.91), estradiol (SMD = −2.72), follicle-stimulating hormone (FSH) (SMD = −7.71), and luteinizing hormone (SMD = −5.54), while increasing malondialdehyde and reducing antioxidant enzymes (SOD, CAT, GPx, and GSH).

**Discussion:**

Flavonoid co-treatment significantly improved hormonal profiles and oxidative balance, with the greatest recovery in FSH. High heterogeneity (I^2^ > 84%) reflected variability in doses, treatment duration, compound purity, and species. Flavonoids exhibit marked ameliorative potential against BPA-induced reproductive toxicity in preclinical models, largely through hormonal regulation and oxidative stress mitigation. Standardized protocols and dose–response studies are essential to enhance reproducibility and translational relevance.

## 1 Introduction

The growing concern about the number of environmental toxins and their impact on the reproductive health of men has been a subject of great interest over the past few years (Mohajer and Culty, 2025). One such chemical is bisphenol A (BPA) (Aja et al., 2024). It is a typical industrial chemical, which can be found in thermal sheets, food containers, and plastics (Hahladakis et al., 2023). It is one of the most outstanding endocrine-disrupting chemicals (EDCs) that are recognized to be detrimental to the reproductive system of both human beings and animals (Cimmino et al., 2020). Bisphenol A (BPA) is an endocrine-disrupting chemical that binds classical estrogen receptors (ERα/ERβ) and other receptors (e.g., GPER, ERRγ, and PPARγ), perturbing downstream transcriptional programs. BPA also triggers organellar and mitochondrial stress with ROS generation, altering redox and metabolic signaling. In parallel, BPA can reprogram epigenetic marks (DNA methylation, histone modifications, and non-coding RNAs), providing a mechanistic link to persistent phenotypes after exposure (Stanojević and Sollner, 2025). Bisphenol A (BPA) has also been repeatedly measured and reported to have acute low toxicity, with reported oral LD_50_ values in rodents in the range of 2,000–5,000 mg/kg (2–5 g/kg) (Agency, 2014). Low doses of BPA may lead to damage to the testicles, disruption of hormones, oxidative stress, and epigenetic alteration, particularly during critical periods of development (Cariati et al., 2019; Prins et al., 2019). A substantial amount of research examining the effect of BPA on reproduction has been conducted with animals (Adegoke et al., 2020; Chianese et al., 2018; Lee et al., 2018; Molangiri et al., 2022). All these models offer a controlled backdrop to investigate the action mode of BPA and long-term effects on male fertility factors, including sperm count, motility, morphology, and hormone levels.

Most plants with therapeutic potential against BPA toxicity contain a group of active metabolites known as flavonoids (Dolatabadi et al., 2025). These flavonoids, a group of non-synthetic polyphenolic compounds, are abundant in several fruits, vegetables, and medicinal herbs and have received substantial research attention due to their antioxidative, anti-inflammatory, and cytoprotective properties (Han et al., 2020). They act as redox-active modulators that upregulate the Nrf2/ARE antioxidant program and dampen NF-κB–driven inflammation, thereby improving cellular stress resilience. They also tune kinase pathways (e.g., MAPK/PI3K-Akt/AMPK) that govern survival and metabolism, and many exhibit context-dependent estrogenic/anti-estrogenic signaling. Several flavonoids additionally exert epigenetic effects (HDAC/DNMT modulation), which may stabilize transcriptional reprogramming of cytoprotective genes (Huang et al., 2023). Identified flavonoids, including quercetin, naringenin, kaempferol, and genistein, have been reported to potentially decrease reproductive system toxicity through multiple mechanisms. This encompasses free radical scavenging, hormonal alteration, and regulating genes (Sirasanagandla et al., 2022). Some of these flavonoids can be phytoestrogens, that is, they can act on estrogen receptors and mimic estrogenic activities (Kiyama, 2023). These facts precondition the dual role of flavonoids in reproductive health as both protective and potentially harmful factors, and the reason why scientific research on the subject is gaining popularity. The dichotomy complicates the process of knowing what their net biological impact may be, particularly when they occur alongside well-established reproductive toxicants such as BPA.

The association of BPA and flavonoids with the reproductive health of men is complex and can be bidirectional. BPA is a xenoestrogen that affects the hypothalamic–pituitary–gonadal (HPG) axis and testosterone synthesis and produces oxidative stress in male reproductive organs, such as the testes and epididymis (Liu et al., 2021; Ryu et al., 2022). Conversely, flavonoids can protect against BPA-induced adverse effects due to their antioxidant properties and their capability to alter the activity of steroidogenic enzymes (Rahman et al., 2019). This presents an alternative pathway to maintain reproductive health. Not all flavonoids, however, act as pure protectants because some are estrogenic at high concentrations or under certain hormonal conditions, which makes it more difficult to consider them as being protective (Kiyama, 2023).

Several studies on animals have yielded different results regarding the impact of flavonoids and BPA on male fertility (Aja et al., 2022; Alboghobeish et al., 2019; Cariati et al., 2019; Sirasanagandla et al., 2022). Some studies show that flavonoids can protect against testicular damage caused by BPA, whereas others report that the benefits are small or not consistent (Bolt and Stewart, 2011). Changes in the study design, such as the species employed (either rat or mouse), the method and length of exposure, the age at which exposure occurred, and the dose, also lead to differences in the results. Because of this inconsistency, it is difficult to draw generalizable conclusions. This means that a full synthesis of all the material that is already available is needed.

An increasing number of people view reproductive health issues, including low sperm quality and testicular dysfunction, as public health concerns, mainly because of environmental and nutritional factors. We need to understand how toxic substances like BPA and potentially protective ones like flavonoids interact to develop public health initiatives, dietary guidelines, and treatment strategies based on data. Also, male reproductive endpoints in rat models are excellent indicators of possible risks to humans, especially because conducting controlled studies on people is difficult (Rahman et al., 2019). Because many individuals are exposed to both BPA and flavonoids daily, this study is highly relevant and necessary (Krivohlavek et al., 2023; Rahman et al., 2021). BPA is common because it is often found in food packaging and industrial products (Krivohlavek et al., 2023). In contrast, flavonoid intake varies widely depending on diet, cultural practices, and use of herbal supplements. To effectively assess and reduce risks, it is important to investigate how these substances impact male reproductive traits both independently and together. This systematic review and meta-analysis aim to synthesize and critically evaluate relevant studies that examine the effects of flavonoids and BPA on male reproductive health, focusing on key fertility parameters such as hormone levels, and assess the extent to which flavonoids mitigate or exacerbate BPA’s reproductive effects on male rodents. More to this, this study will help identify gaps in the literature and methodological limitations that may influence the interpretation of findings and guide future research.

## 2 Methodology

### 2.1 Article search

A comprehensive literature search was systematically conducted across the Web of Science (WoS), Scopus, and PubMed databases on 16 September 2024. The search employed the following terms: “Bisphenol A,” “flavonoids,” “male rodents,” and “East Africa.” Boolean operators (AND/OR/NOT), alternative terms, and various delimiters such as quotation marks, parentheses, wildcards, and asterisks (*) were utilized to create the search strategy outlined in Table 1, as reported by Fasogbon et al. (2023), Fasogbon et al. (2024), and Fasogbon et al. (2025). The search was restricted to peer-reviewed English-language articles. The article selection process adhered to the Preferred Reporting Items for Systematic Reviews and Meta-Analyses (PRISMA) 2020 guidelines (Page et al., 2021; Swase et al., 2025).

**TABLE 1:** Search strategies.

Database	Search strategy
Scopus	TITLE-ABS-KEY (“Bisphenol A” OR “Bisphenol-A” OR BPA OR BP-A) AND (flavonoid* OR naringin OR nariginin OR hesperidin) AND (reproduc* OR *fertilit* OR sperm* OR hormon*) AND (male) AND (rodent* OR mice OR mouse OR rat* OR rabbit*)
WoS	(“Bisphenol A” OR “Bisphenol-A” OR BPA OR BP-A) AND (flavonoid* OR naringin OR nariginin OR hesperidin) AND (reproduc* OR *fertilit* OR sperm* OR hormon*) AND (male) AND (rodent* OR mice OR mouse OR rat* OR rabbit*) TOPIC
PubMed	(((“Bisphenol A” [Title/Abstract]) OR (Bisphenol-A [Title/Abstract])) AND ((flavonoid*[Title/Abstract]) OR (hesperidin [Title/Abstract]) OR (naringin [Title/Abstract]) OR (nariginin [Title/Abstract]) OR (quercetin [Title/Abstract]) OR (apigetrin [Title/Abstract])) AND ((reproduc*[Title/Abstract]) OR (fertility [Title/Abstract]) OR (infertility [Title/Abstract])OR (sperm*[Title/Abstract]) OR (hormon*[Title/Abstract])) AND (male [Title/Abstract]) AND ((rodent*[Title/Abstract]) OR (mice [Title/Abstract]) OR (mouse [Title/Abstract]) OR (rat [Title/Abstract]) OR (rabbit*[Title/Abstract])) NOT (review [Publication Type]))

### 2.2 Inclusion and exclusion criteria

To ensure methodological rigor and relevance to the research question, we applied well-defined inclusion and exclusion criteria based on the PICOS framework (Population, Intervention/Exposure, Comparison, Outcome, Study Design). These criteria were applied consistently during the title, abstract, and full-text screening phases. Only studies meeting all inclusion criteria and free from exclusion factors were advanced for final analysis. The criteria were tailored to capture original research reporting BPA toxicity in male rodents and the ameliorative effect of flavonoids, as detailed in Table 2.

**TABLE 2:** Inclusion and exclusion criteria.

Category	Inclusion	Exclusion
Population	Studies involving male rodents (e.g., mice, rats, rabbits)	Studies involving female rodents or non-rodent species
Intervention	Studies on the toxic effects of bisphenol-A (BPA) and the protective role of flavonoids (e.g., naringin, naringenin, hesperidin, quercetin, and apigetrin)	Studies that do not involve flavonoids or focus on unrelated compounds or exposures
Outcomes	Studies reporting reproductive health outcomes: sperm quality, hormone levels, fertility, etc.	Studies that do not assess reproductive outcomes or focus on unrelated health aspects
Study Type	Original research articles, including experimental and observational studies	Reviews, meta-analyses, editorials, letters, conference abstracts, and non-original research
Language	Published in English	Published in non-English languages
Publication Type	Articles from peer-reviewed journals	Grey literature, theses, dissertations, or unpublished studies

### 2.3 Data extraction

Following the article eligibility determination, a standardized data extraction tool was developed to ensure consistency and completeness in capturing relevant study details. The tool was pilot-tested on a sample of included studies and refined for clarity and efficiency. It included bibliographic details, BPA exposure details, flavonoid intervention details, and key findings. This framework enabled structured synthesis and comparative interpretation of data across studies. All extracted variables are summarized in Table 3 below.

**TABLE 3:** Data to be extracted from all included articles.

Category	Field
Study characteristics	Author(s)
	Year of publication
	Study location
Exposure details	Type of flavonoid administered
	Dosage of flavonoid
	Duration of flavonoid administration
Intervention details	Dosage of bisphenol-A (BPA)
	Duration of BPA exposure
	Type, dosage, and duration of flavonoid co-treatment (if applicable)
Outcomes measured	Specific reproductive health outcomes (e.g., sperm count, motility, and testosterone levels)
	Methods used to assess outcomes (e.g., ELISA, microscopy, CASA, and histology)
Results	Key findings related to reproductive health
	Statistical significance of results (e.g., p-values and confidence intervals)
	Any reported side effects or adverse outcomes
Quality assessment	Study limitations (e.g., sample size, methodological flaws, and risk of bias)

### 2.4 Risk of bias analysis

**FIGURE 1:**
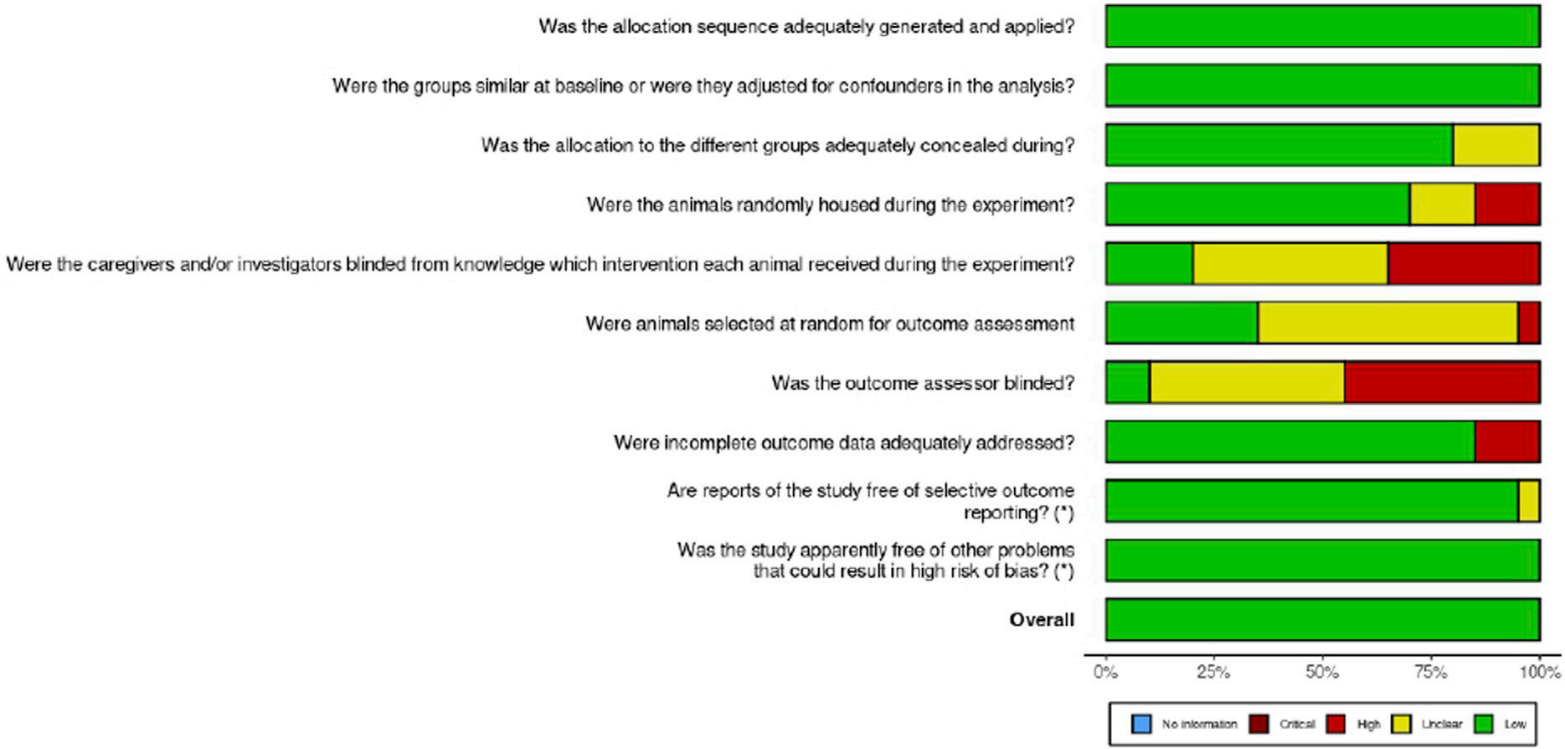
Risk of bias for the various domains.

**FIGURE 2:**
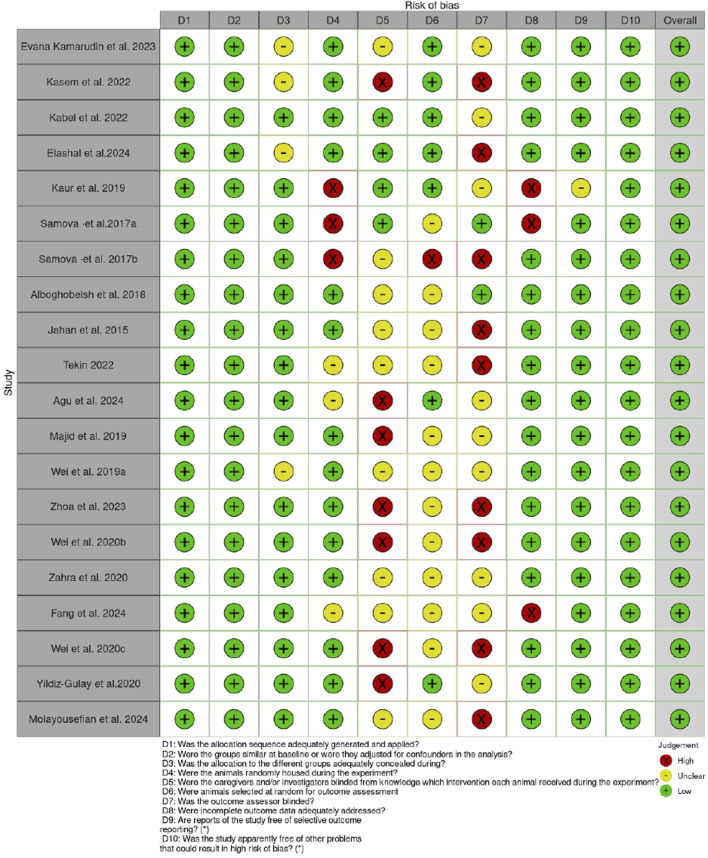
Risk of bias assessment for the individual articles.

The Systematic Review Center for Laboratory Animal Experimentation (SYRCLE) Checklist for *in vivo* research was used to assess the risk of bias (Hooijmans et al., 2014). The ROBVIS (Risk-Of-Bias VISualization) tool was used to present the outcome of the risk of bias assessment, as shown in Figures 1, 2.

### 2.5 Pooling of data and assessment of heterogeneity

Data on variables reported on at least six studies were pooled. To combine or merge the effect sizes in hormonal and oxidative stress outcomes, the standardized mean difference (SMD) was computed, as these measures were on a ratio scale. To measure other variables, Hedges g (a bias-corrected SMD) was employed in measuring the significance of the effect size. Random-effects models were implemented, and restricted maximum likelihood (REML) estimation was used to estimate the variance between studies and to produce pooled effect sizes of each outcome. Pooled estimates were adjusted into 95% confidence intervals (CIs) according to Knapp and Hartung (2003). The findings of the meta-analysis were also summarized into forest plots to portray them visually. A sensitivity analysis was performed to measure the sensitivity of the results by re-running the analysis using a fixed-effect model and leave-one-out procedure. Tau 2 and Cochran Q statistic (Q^2^) and its p-value measure between-study variance, and the percentage of residual variation explained by the heterogeneity is I^2^. Each analysis has been done using Comprehensive Meta-Analysis (CMA) software 4, and all p-values are set at less than 0.05.

## 3 Results

### 3.1 Search results

**FIGURE 3:**
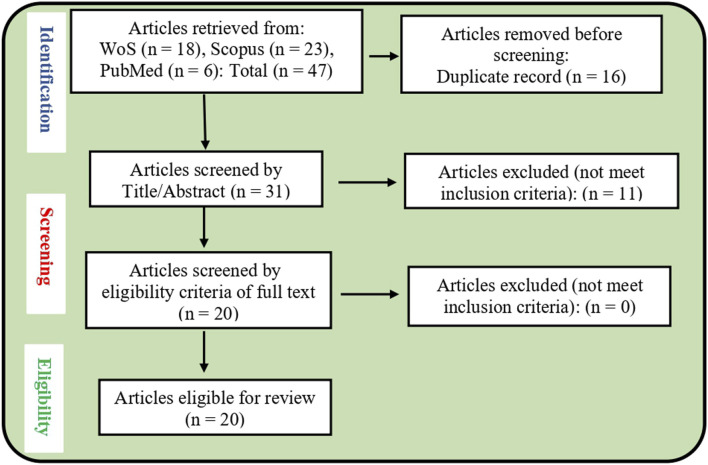
PRISMA study selection flow chart.

The Web of Science, Scopus, and PubMed databases yielded 18, 23, and 6 articles, respectively, bringing the total to 47 articles across the three databases. The search results from each database were then exported and imported into Rayyan, a platform specifically designed for systematic review processes, where they were screened based on the inclusion and exclusion criteria (Johnson and Phillips, 2018). On the platform, 16 duplicate articles were removed. The remaining 31 records were initially screened by evaluating their titles and abstracts, which led to the exclusion of 11 articles. No additional articles were excluded following a more detailed full-text review because all 20 articles met the inclusion criteria. Therefore, 20 articles were included in the study after passing the eligibility criteria and quality assessment (Figure 3).

### 3.2 Utilization of plant-derived flavonoids in BPA exposure

Table 4 summarizes a wide view of plant-derived bioactive compounds and the evidence for their experimental uses in mitigating BPA-induced toxicity in male rodents. The table collates studies employing both pure flavonoid compounds (e.g., quercetin, naringin, hesperidin, and kaempferol) and crude plant extracts that are rich in flavonoids and other phytochemicals.

**TABLE 4:** Plant-derived bioactive compounds and their experimental application in animal models with BPA exposure.

S/No	Plant names	Plant part used	Type of extract	Bioactive compound	Class of bioactive compounds	Number/animal model used	Route of administration	Flavonoid dose administered in mg/kg	BPA dose administered in mg/kg	Period of study (in days)	Reference
1	Silk fibroin	NA	Aqueous extract		Icariin	30/Male mice	Oral	50 mg/kg	BPA (50 mg/kg/d)	60 days	Fang et al. (2024)
2	*Hyllanthus gomphocarpus Hook. F*	Root	Aqueous extract		Phenolic and flavonoid	24/Male rats	Oral	75 mg/kg	1 mL of 200 mg/kg of BPA	21 days	Kamarudin et al. (2023)
3	NA	NA		Pure sample (hesperidin)	Flavanone glycoside	42/Male rats	Oral	200 mg/kg	160 mg/kg	60 days	Kasem et al. (2022)
4	NA	NA		Pure sample (fluvastatin and taxifolin)	Fluvastatin and taxifolin	48/Male rats	Oral gavage	10 mg/kg/day and 50 mg/kg/day	BPA (20 μg/kg)	60 days	Kabel et al. (2022)
5	Apilarnil	NA	NA		Lysine, apigenin 7-glucoside, flavonoids, 10-hydroxy-(E)-2-dodecenoic acid, and campesterol	48/Male rats	Orally	0.6 g/kg bw	BPA (50 mg/kg/body weight)	45 days	Elashal et al. (2024)
6	*Murraya koenigii*	Leaves	Hydroethanolic		Tannins, alkaloids, flavonoids, proteins, steroids/polysterols, glycosides, carbohydrates, and triterpenoids	30/Male mice	Orally	200 mg/kg body weight	BPA at a dose level of 1 mg/kg body weight	60 days	Kaur et al. (2020)
7		NA	Pure sample	Quercetin	NA	90/Male mice	Orally	30 mg/kg, 60 mg/kg, and 90 mg/kg body weight	BPA at 80 mg/kg, 120 mg/kg, and 240 mg/kg	45 days	Samova et al. (2008)
8		NA	Pure sample	Quercetin	NA	90/Male mice	Orally	90 mg/kg bodyweight/day	80 mg/kg, 120 mg/kg, and 240 mg/kg body of BPA	45 days	Samova et al. (2008)
9		NA	Pure sample	Naringin	NA	36/Male rats	Orally	160 mg/kg	1 ml of BPA (50 mg/kg body weight)	30 days	Alboghobeish et al. (2019)
10		NA	Pure sample	Quercetin	NA	20/Male rats	Orally	50 mg/kg	50 mg/kg of BPA	52 days	Jahan et al. (2016)
11		NA	Pure sample	Hesperidin	Flavonoid	52/Male rats	Orally	50 mg/kg	100 mg/kg of BPA	14 days	Tekin and Çelebi (2022)
12		NA	Pure sample	*Cuscuta chinensis*	Flavonoids	100/Male and female mice	Orally	20 mg/kg, 30 mg/kg, and 40 mg/kg	BPA at 5 mg/kg/day	74 days	Wei et al. (2020)
13	Punicalagin	NA	NA		Polyphenols	24/Male rabbits	Orally	2 mg/kg	BPA (20 mg/kg live weight)	63 days	Yildiz-Gulay et al. (2020)
14		NA	Pure sample	Kaempferol	Flavonoids	35/Male rats	Orally	250 mg/kg	10 mg/kg BPA	35 days	Molayousefian et al. (2024)
15	*Cucumeropsis mannii*	Seeds	Crude extract		Flavonoids	24/Male rats	Orally	7.5 mL/kg	100 mg/kg body weight (BW) of BPA	63 days	Agu et al. (2024)
16	*Ipomoea batatas L. Lam*	Plant tubers and veins	Crude extract		Phenols and flavonoids, coumarins and saponins, terpenoids and triterpenoids, tannins and quinones, anthocyanin, and betacyanin	112/Male rats	Orally and intraperitoneally	300 mg/kg	BPA (50 mg/kg)	21 days	Majid et al. (2019)
17		NA	Pure sample	*Cuscuta chinensis* flavonoids	Flavonoids	100/Pregnant female mice and 5 male mice	Orally	120 mg/kg	BPA at 5 mg/kg	73 days	Wei et al. (2019)
18		NA	Pure sample	*Cuscuta chinensis* flavonoids	Quercetin, kaempferol, kaempferol-3-O-β-D-glucopyranoside, Isorhamnetin-3-O-β-D-glucopyranoside, hyperoside, trans-tiliroside, quercetin-3-O-(6″-feruloyl)-β-D-galactopyranoside, kaempferol-3-O-(2″-O-E-p-coumaroyl)-β-D-glucopyranoside, kaempferol-3-O-β-D-(6″-p-hydroxybenzoyl)-galactopyranoside, kaempferol-3-O-(6″-O-E-feruloyl)-β-D-glucopyranoside	45/Pregnant female mice and 3/male mice	Orally	40 mg/kg bw	5 mg/kg bw of BPA	56 days	Zhao et al. (2023)
19		NA	Pure sample	*Cuscuta chinensis* flavonoids	Flavonoids	100/pregnant female mice and 5/male mice	Orally	40 mg/kg	5 mg/kg of BPA	74 days	Wei et al. (2020)
20	*Vincetoxicum arnottianum*	Aerial parts	Methanol extract		Total phenolic, flavonoid, alkaloid, β-carotene, and lycopene	56/male rats	Orally	150 mg/kg	25 mg/kg of BPA	30 days	Zahra et al. (2020)

### 3.3 Percentage geographic distribution of included studies and phytochemical analysis method reported

**FIGURE 4:**
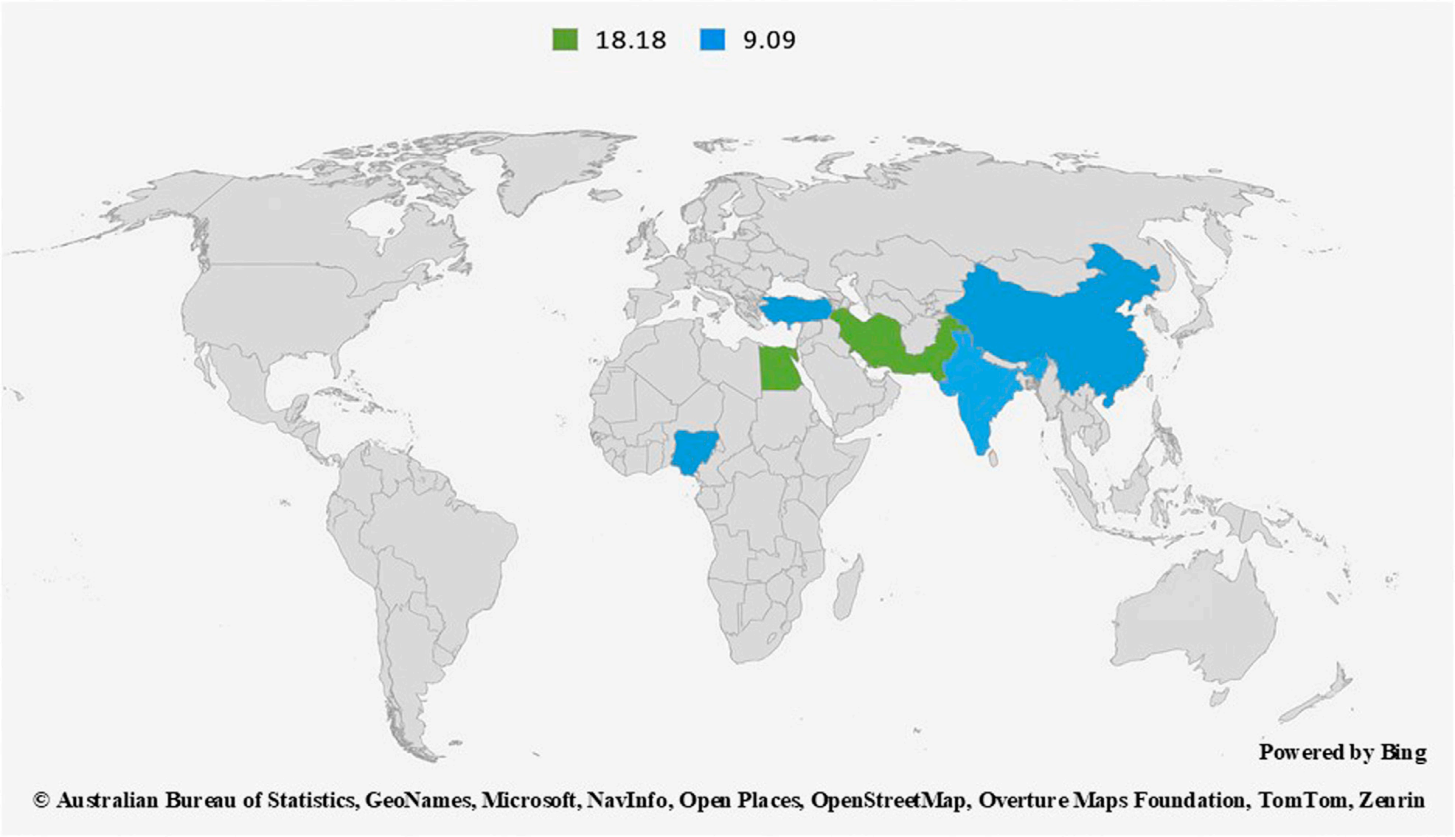
Geographic distribution of study contributions by country (%).

**FIGURE 5:**
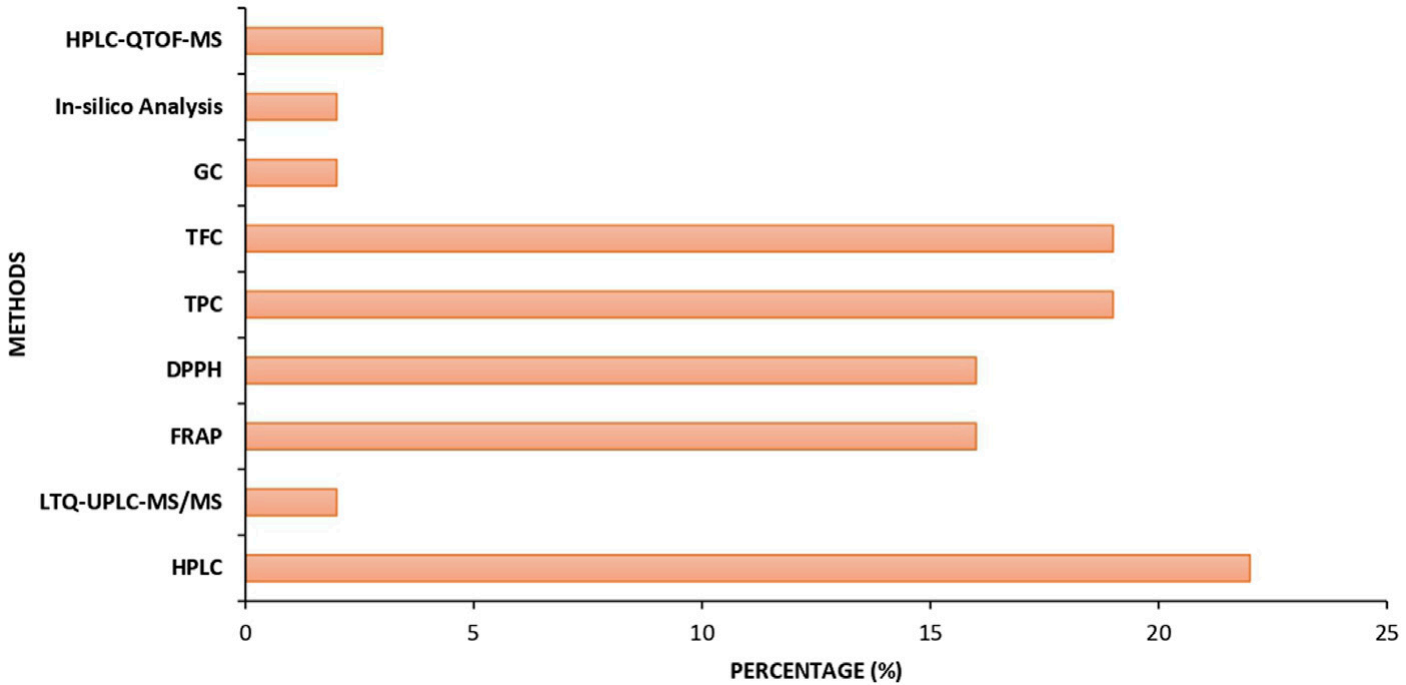
Percentage of phytochemical analysis methods used in studies.

The data presented in Figure 4 illustrate the regional contributions to research on the use of flavonoids in mitigating BPA-induced toxicity. The figure reveals that a substantial proportion of the studies were conducted in China, followed by India, Iran, Egypt, and Nigeria. Additionally, Figure 5 depicts the percentage distribution of phytochemical analysis methods such as high-performance liquid chromatography quadrupole time-of-flight mass spectroscopy (HPLC-QTOF-MS), *in silico* analysis, gas chromatography (GC), total flavonoid content (TFC), total phenolic content (TPC), 2,2-Diphenyl-1-picrylhydrazyl (DPPH), ferric reducing antioxidant power (FRAP), linear trap quadupole-ultra-high performance liquid chromatography-mass spectroscopy (LTQ-UPLC-MS/MS), and HPLC employed in the reviewed studies. Notably, HPLC dominates the phytochemical analysis approaches reported in the study.

### 3.4 Effect of flavonoid on hormonal imbalance induced by BPA

**FIGURE 6:**
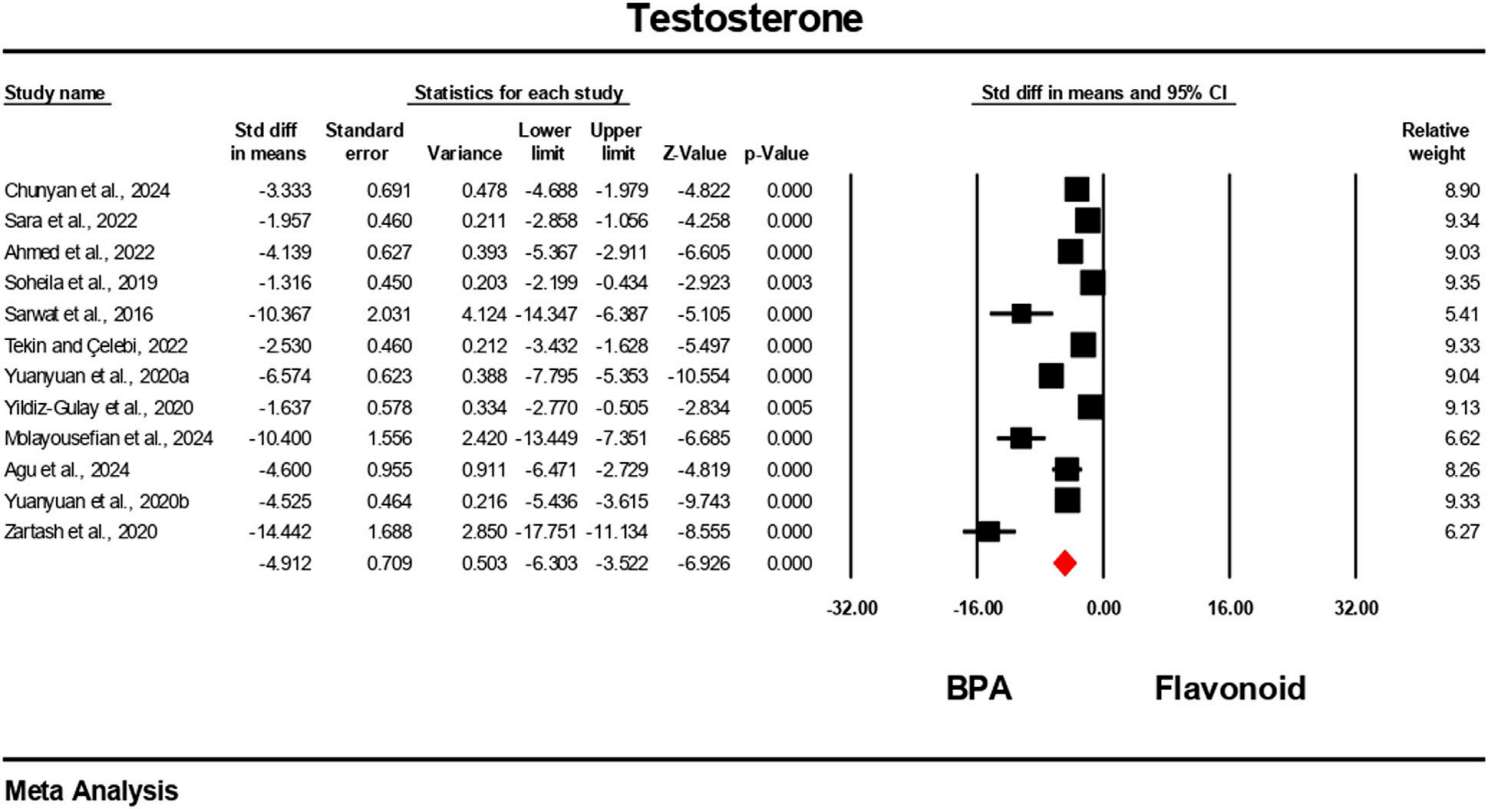
Interplay of the flavonoid on testosterone levels following BPA exposure.

**FIGURE 7:**
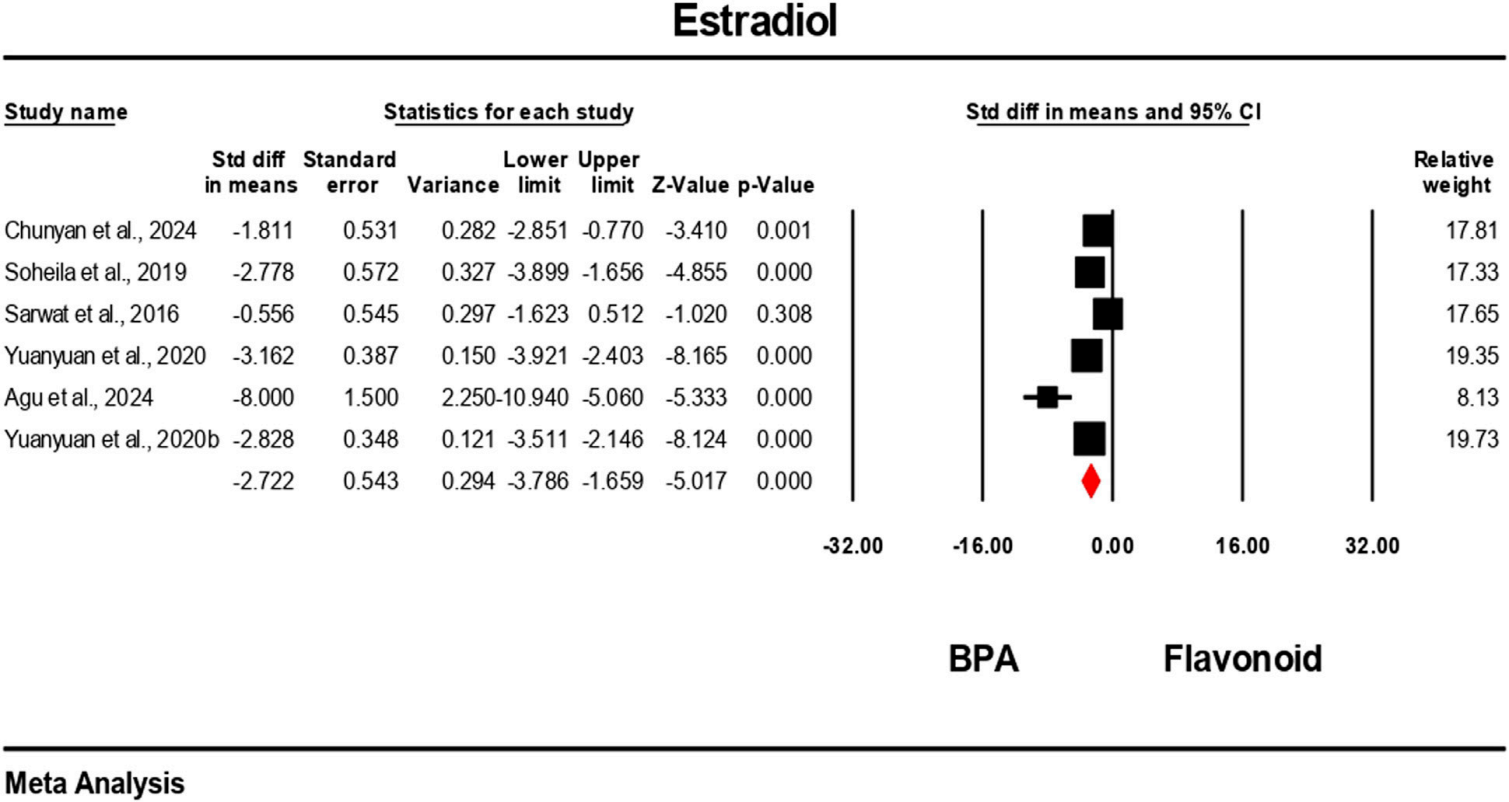
Interplay of the flavonoid on estradiol levels following BPA exposure.

**FIGURE 8:**
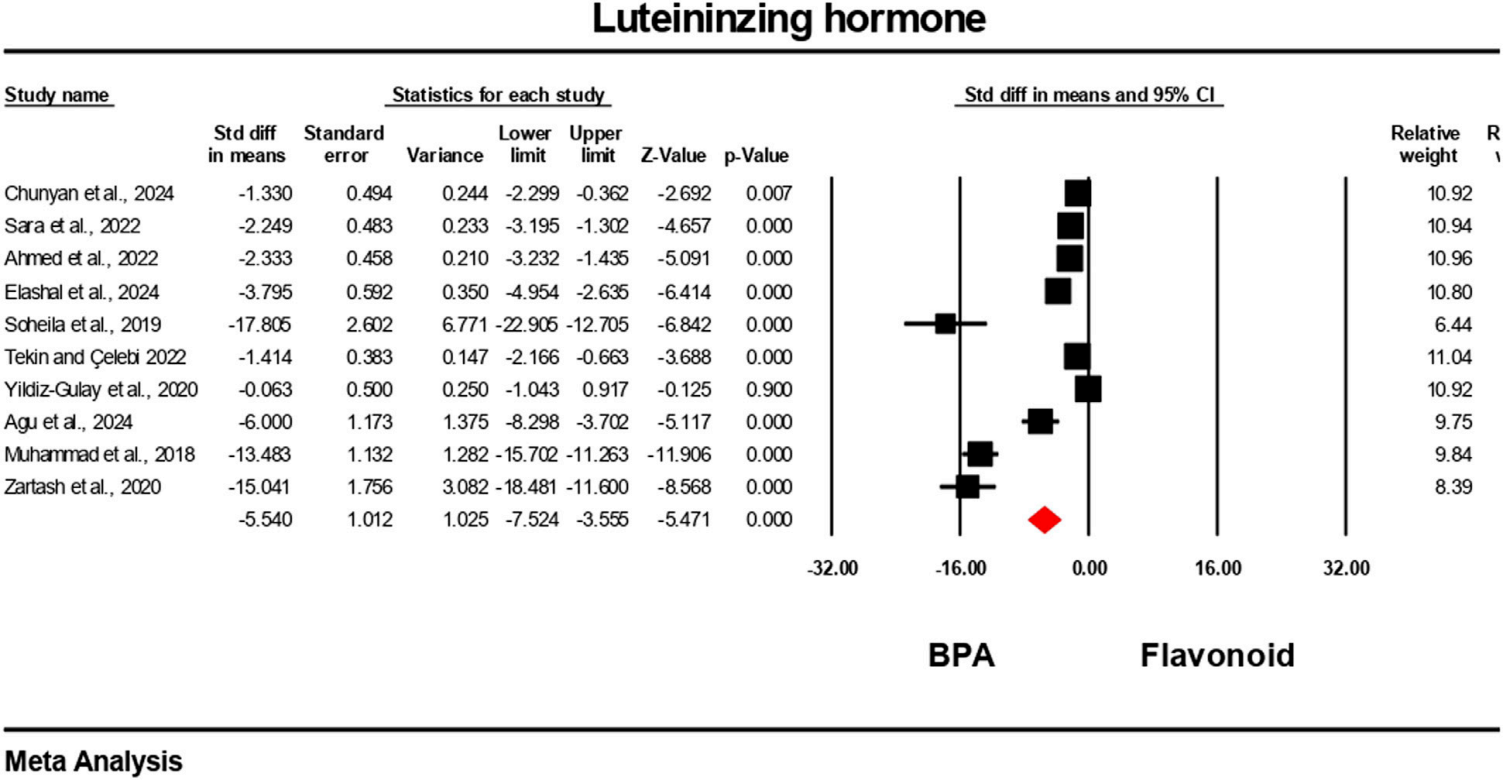
Interplay of the flavonoid on follicle-stimulating hormone levels following BPA exposure.

**FIGURE 9:**
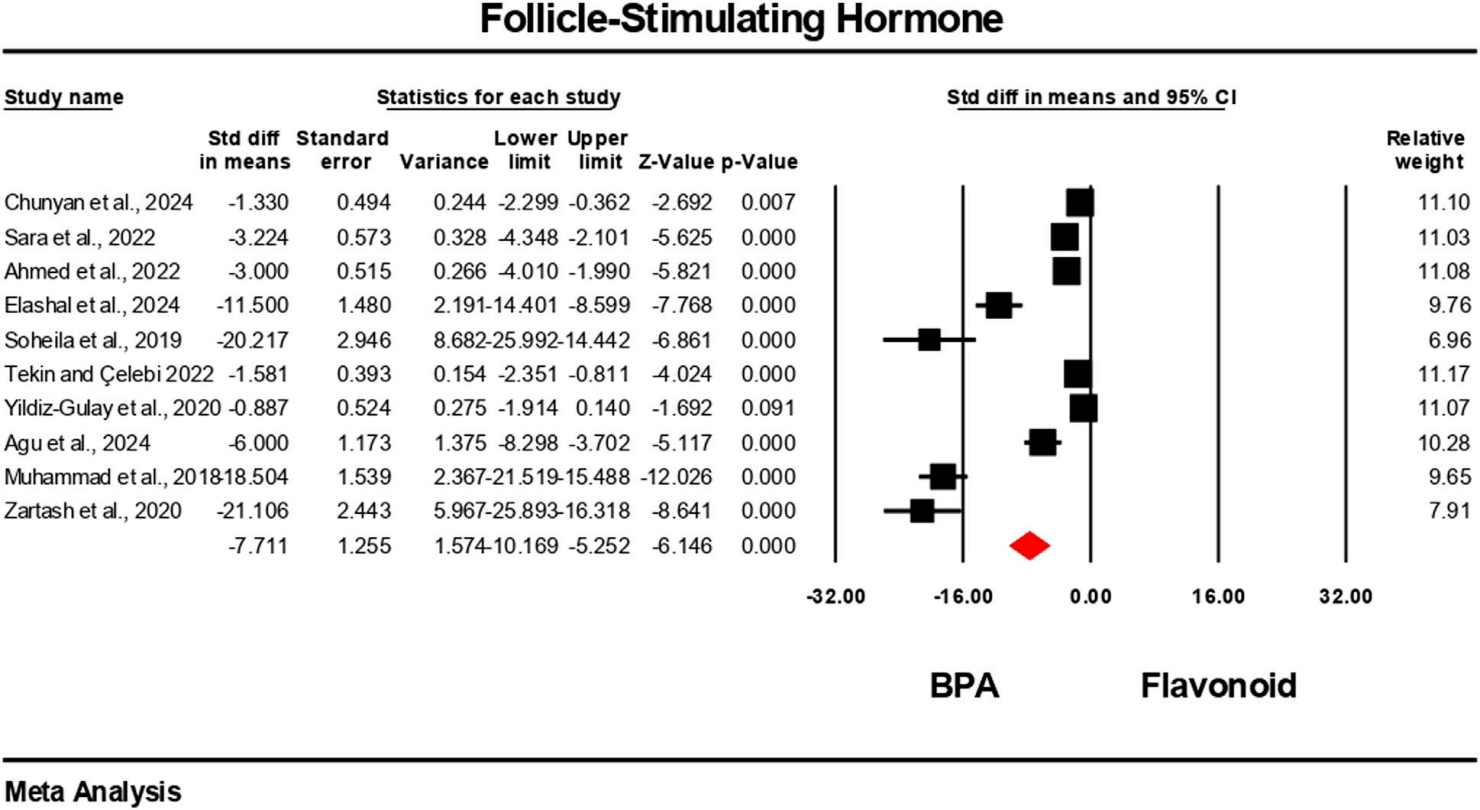
Interplay of the flavonoid on luteinizing hormone levels following BPA exposure.

Hormonal level markers for both the BPA and flavonoid-treated groups were reported in at least six independent studies. Data were pooled using a random-effects model. Compared to the flavonoid treatment group, the BPA-only treated group experienced a significant decrease in the levels of testosterone, FSH, luteinizing hormone, and estradiol. The estimated pooled effect sizes were as follows: testosterone (standardized mean difference [SMD] = −4.912, 95% CI: 6.303 to −3.522, p = 0.0001); estradiol (SMD = −2.722, 95% CI: 3.786 to −1.659, p = 0.0001); FSH (SMD = −7.711, 95% CI: 10.169 to −5.252, p = 0.0001); and luteinizing hormone (SMD = −5.540, 95% CI: 7.524 to −3.555, p = 0.0001). The analysis revealed statistically significant heterogeneity among the individual studies for all hormonal markers. Heterogeneity statistics were as follows: testosterone: I^2^ = 92.84%, Tau^2^ = 5.178, Q = 153.690, df = 11, p = 0.0001; estradiol: I^2^ = 84.30%, Tau^2^ = 1.372, Q = 31.841, df = 5, p = 0.0001; FSH: I^2^ = 96.66%, Tau^2^ = 13.939, Q = 269.723, df = 9, p = 0.0001; luteinizing hormone: I^2^ = 96.10%, Tau^2^ = 9.140, Q = 230.913, df = 9, p = 0.0001. These results indicate a very high and statistically significant level of heterogeneity (p < 0.05) across the included studies for all hormonal parameters (Figures 6–9).

### 3.5 Effect of flavonoid on BPA-induced oxidative stress level

**FIGURE 10:**
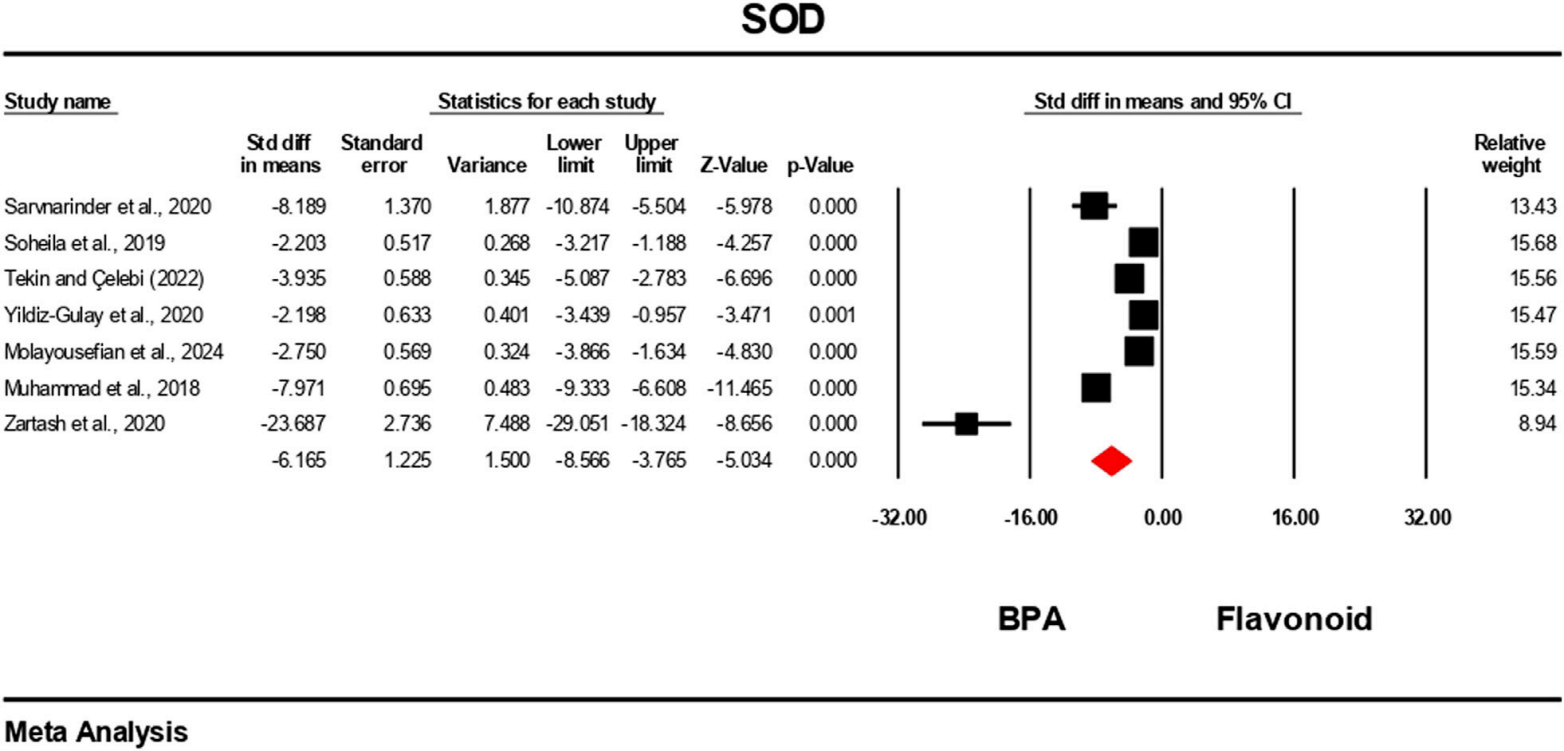
Interplay of the flavonoid on SOD levels following BPA exposure.

**FIGURE 11:**
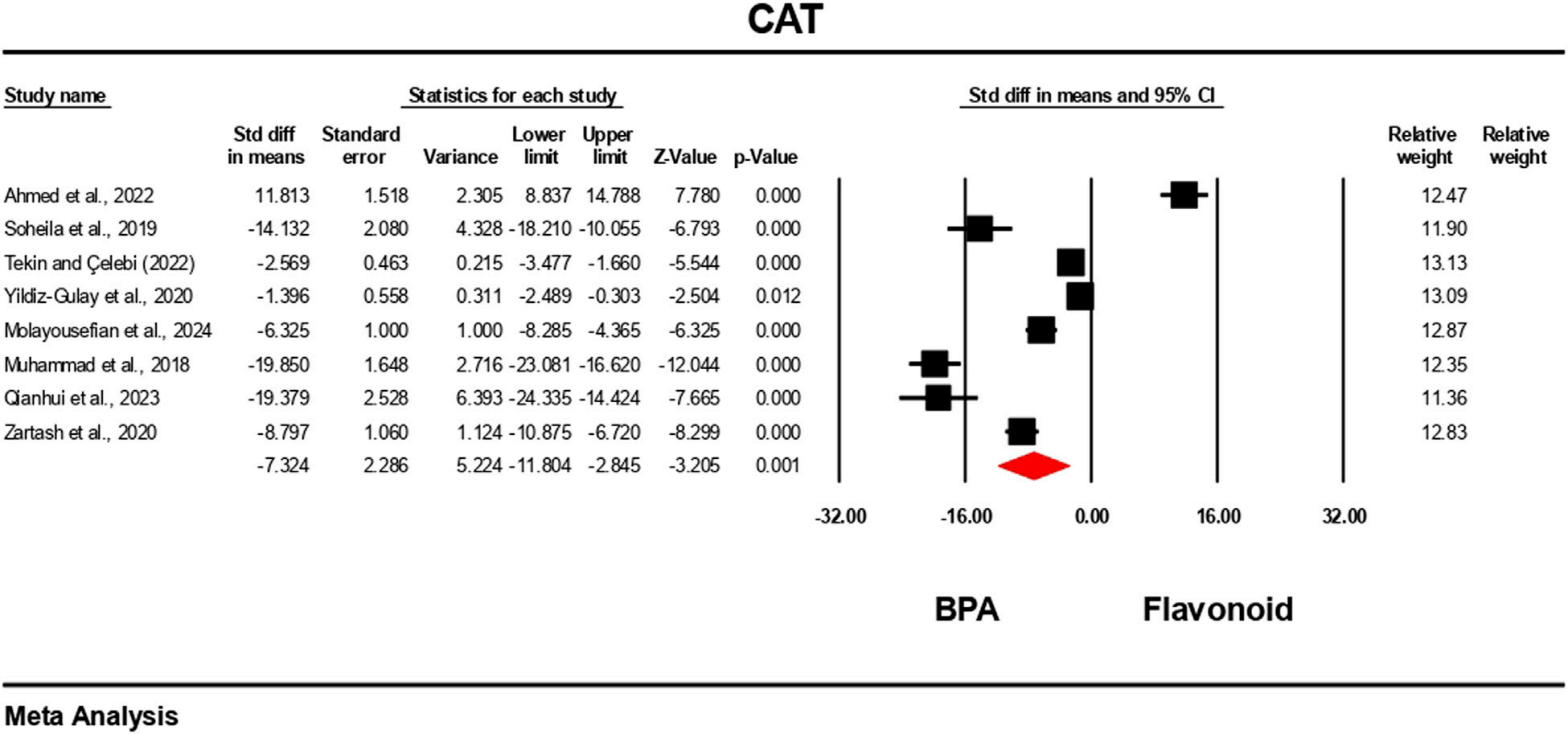
Interplay of the flavonoid on CAT levels following BPA exposure.

**FIGURE 12:**
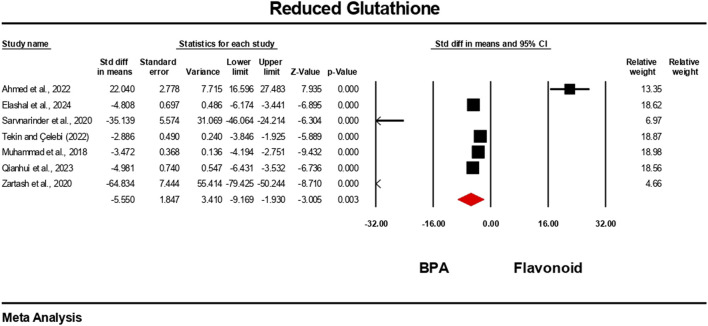
Interplay of the flavonoid on reduced glutathione levels following BPA exposure.

**FIGURE 13:**
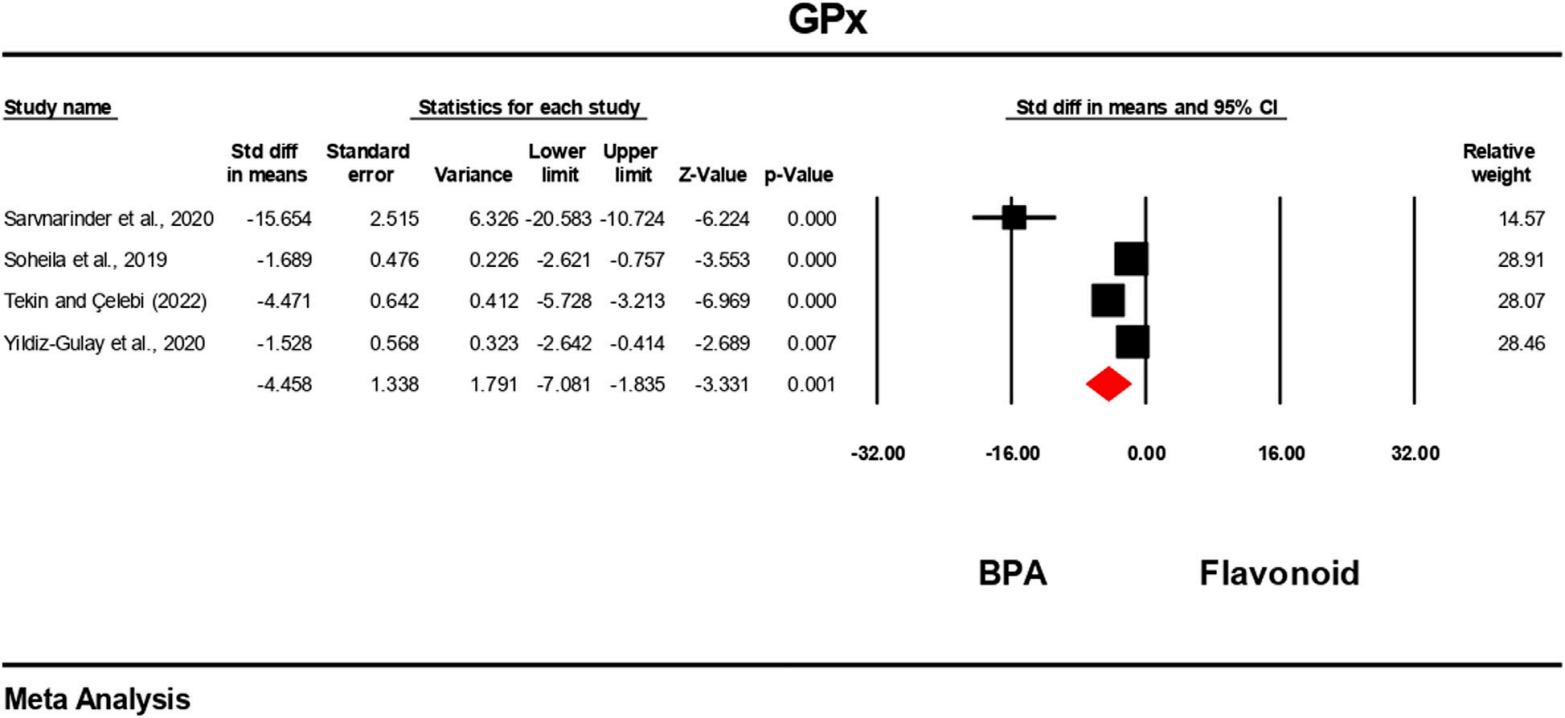
Interplay of the flavonoid on GPx levels following BPA exposure.

**FIGURE 14:**
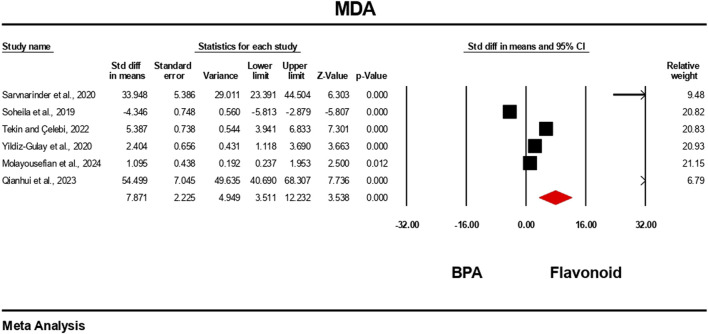
Interplay of the flavonoid on MDA levels following BPA exposure.

The oxidative stress level markers were measured for both the BPA and flavonoid-treated groups in at least five independent studies. The random-effects model was used to bring the data together. When evaluated against the flavonoid treatment group, the group treated with BPA alone showed decreased antioxidant levels because the levels of SOD, CAT, GPx, and GSH decreased (Figures 10–13), while the level of malondialdehyde (MDA) increased significantly (Figure 14). According to the results, SOD showed a negative pooled effect size of −8.043 (95% CI: 10.797 to −5.289, p = 0.001), CAT had a negative pooled effect size of −7.324 (95% CI: 11.804 to −3.205, p = 0.001), and GPx had a negative pooled effect size of −4.458 (95% CI: 7.081 to −1.835, p = 0.001). All the oxidative stress markers showed high heterogeneity in the individual studies. Heterogeneity statistics for SOD were SOD: I^2^ = 95.818%, Tau^2^ = 13.221, Q = 167.398, df = 7, p = 0.0001; for CAT were I^2^ = 97.791%, Tau^2^ = 39.582, Q = 316.874, df = 7, p = 0.0001; for GPx were I^2^ = 92.959 This means that the studies included a very high level of variation (p < 0.05) for all measures of oxidative stress (Figures 10–14).

## 4 Discussion

This systematic review and meta-analysis evaluated the effects of bisphenol A (BPA) and flavonoids on male reproductive health in rodent models. These studies utilized various natural compounds, such as quercetin (Jahan et al., 2016; Samova et al., 2018), naringin (Alboghobeish et al., 2019), kaempferol (Molayousefian et al., 2024), hesperidin (Kasem et al., 2022; Tekin and Çelebi, 2022), punicalagin (Yildiz-Gulay et al., 2020), taxifolin (Kabel et al., 2022), and crude plant extracts (Fang et al., 2024; Kamarudin et al., 2023), administered either alone or in combination with BPA to assess protective or mitigating effects on reproductive endpoints. The findings stressed the complex relationship between exogenous toxicants like BPA and dietary polyphenols such as flavonoids, providing a better understanding of their potential antagonistic or synergistic effects on key reproductive outcomes. The experimental models covered both rats and mice, with oral administration being the dominant route. BPA doses ranged widely, from as low as 1 mg/kg (Alboghobeish et al., 2019; Kaur et al., 2020) to as high as 240 mg/kg (Samova et al., 2008, 2018), with duration spanning 14 days (Tekin and Çelebi, 2022) to 74 days (Wei et al., 2020). This diversity in dose, duration, and delivery route reflects the heterogeneity in methodological approaches across the literature. This has implications for reproducibility and comparability of outcomes (Harden and Thomas, 2005).

The quality assessment using the SYRCLE checklist and ROBVIS (Risk-Of-Bias VISualization) tool revealed that although some domains, such as baseline comparability and incomplete outcome data, were adequately addressed in most studies, others, including sequence generation, allocation concealment, and blinding, were not well reported. In general, the findings indicate that the selected studies are informative and can guide the research question, although some risks of bias can limit the robustness of the findings. However, the reliability of the results between the studies is high enough, and the interpretation should be approached with consideration. The major limitation is the failure to report all methodological details, as this may conceal the actual risk of bias. It would be desirable in the future to follow standardized reporting guidelines such as ARRIVE 2.0, utilize a more rigorous randomization and blinding procedure, and provide a clear and detailed report. Such enhancements will increase reproducibility, minimize possible bias, and eventually raise the trust in the outcomes of preclinical studies.

### 4.1 Experimental and geographic trends

Analysis of the trend of study experimental methodologies showed a dominant reliance on oral gavage, which aligns with typical dietary exposure routes, indicating a dominant reliance on oral gavage for both BPA and flavonoid administration. This is appropriate for mimicking dietary or environmental exposure routes. Only a minority of studies used intraperitoneal injection, such as Majid et al. (2019), a route that may significantly alter compound pharmacokinetics (de Bree et al., 2017; Dedrick and Flessner, 1997). The experimental trend also suggests that most studies employed histopathological and biochemical endpoints, but fewer included behavioral or transgenerational analyses, representing a gap in research. The geographic distribution of study contributions by country reveals that a disproportionate number of studies were conducted in South and East Asian countries. While these regions have contributed substantially to the field, the geographic skew limits external validity. The dietary culture of different regions, environmental BPA levels, and genetic differences in rodent strains may influence findings. For instance, certain dietary flavonoids are more commonly available in Asian than in African diets, which could affect baseline oxidative status and hormone levels. Hence, expanding research to include African, European, and American cohorts would improve the generalizability of findings. Moreover, only a few studies, such as Zahra et al. (2020) and Agu et al. (2024), originate from African contexts, despite growing BPA exposure in developing countries due to unregulated plastic usage. Additionally, looking at the phytochemical analysis methods employed in the reviewed studies, high-performance liquid chromatography (HPLC) was found to be the most frequently used phytochemical analysis method across the included studies, which may indicate a strong preference for high-resolution, quantitative methods capable of accurately identifying and measuring individual flavonoid compounds (Nassarawa et al., 2024). This preference reflects a growing emphasis on precision and compound specificity in BPA toxicity research, although the other methods used in some studies may have been selected due to their simplicity and affordability. The prevalence of HPLC demonstrates a methodological shift toward more reliable and standardized analytical techniques, which is crucial for comparing results across different studies and ensuring reproducibility.

### 4.2 Effects of flavonoids and bisphenol A on hormonal regulation

Pooled data from previous studies suggest that exposure to BPA resulted in significant disruption of endocrine function, as demonstrated by alterations in testosterone, FSH, LH, and estradiol levels (Aja et al., 2024; Akingbemi et al., 2004; Fernández et al., 2009; Miao et al., 2015). This hormonal disruption may likely be as a result of BPA’s interfering with the hypothalamic–pituitary–gonadal (HPG) axis and its activity in estrogen production (Xue et al., 2024). The consistent hormonal perturbations observed across multiple studies reinforce BPA’s classification as a potent endocrine-disrupting chemical (Meng et al., 2023; Zhang et al., 2023). The meta-analysis found substantial improvements in these hormone levels following flavonoid co-treatment. The strongest effect size was observed in FSH, followed by LH, testosterone, and estradiol; all were statistically significant. These findings are in line with individual studies. For instance, several studies demonstrated that quercetin reversed BPA-induced testosterone decline in rats (Jahan et al., 2016; Samova et al., 2008, 2018), showing dose-dependent improvements in sperm quality and hormonal regulation. Similarly, hesperidin ameliorated estradiol and FSH dysregulation in rats (Kasem et al., 2022), confirming the role of flavonoids in modulating the hypothalamic–pituitary–gonadal axis. Several studies in Table 4 demonstrate the ameliorative effects of flavonoids against BPA-induced toxicity. For instance, icariin, derived from silk fibroin, showed comparable protective effects by reducing testicular oxidative stress and improving hormonal balance (Fang et al., 2024). Other studies incorporated complex phytochemical mixtures. For example, *Ipomoea batatas* extract, rich in phenols, flavonoids, saponins, and terpenoids, improved antioxidant status in rats exposed to 50 mg/kg BPA (Majid et al., 2019). Similarly, *Murraya koenigii* leaf extract, containing tannins, steroids, and flavonoids, mitigated testicular histological damage in mice (Kaur et al., 2020).

### 4.3 Flavonoid and bisphenol A effects on oxidative stress pathways

Oxidative stress is a central mechanism through which BPA exerts toxicity, particularly in reproductive tissues (Meli et al., 2020; Sharma et al., 2019). The meta-analysis of five studies evaluated antioxidant enzymes, SOD, CAT, GPx, and GSH, and the lipid peroxidation marker MDA. Contrary to expectations, levels of antioxidant enzymes were significantly reduced in flavonoid-treated groups compared to BPA-alone groups, while MDA increased. At the first glance, these results seem to contradict established findings that flavonoids exert antioxidant effects (Speisky et al., 2022; Zahra et al., 2024). However, the paradox may reflect methodological limitations or tissue-specific responses. When administered in high doses, some flavonoids exert a pro-oxidant effect, depending on redox status and enzymatic interactions (Barrett, 1996; Jomova et al., 2025). Nevertheless, individual studies such as Fang et al. (2024) and Agu et al. (2024) reported positive antioxidant effects in testicular tissue, suggesting that flavonoid efficacy may be organ-dependent. Interestingly, flavonoid-rich compounds like hesperidin and taxifolin (Kabel et al., 2022; Kasem et al., 2022) demonstrated protective effects even at moderate BPA doses, suggesting their strong antioxidant and hormonal regulatory potential, which might have restored hormonal homeostasis by attenuating oxidative damage and modulating steroidogenic enzymes (Alharbi et al., 2025; Ferraz et al., 2020).

### 4.4 Methodological heterogeneity

High levels of heterogeneity (I^2^ > 84%) were observed across all hormonal and oxidative stress outcomes. Factors contributing to this include variation in BPA dosage, treatment duration, flavonoid type, purity and concentration, animal species, and administration routes, as well as inconsistencies in hormonal assay techniques. For example, BPA doses ranged from as low as 1 mg/kg (Kaur et al., 2020; Kaur and Sadwal, 2020) to as high as 240 mg/kg (Samova et al., 2018), and treatment durations varied from 14 days (Tekin and Çelebi, 2022) to 74 days (Wei et al., 2020). Studies employing pure compounds such as hesperidin or taxifolin reported more pronounced effects than those using crude plant extracts, suggesting that compound standardization is crucial for reproducibility (Kabel et al., 2022; Kasem et al., 2022), highlighting the importance of phytochemical standardization. Kabel et al. (2022) employed a combined treatment of taxifolin and fluvastatin, complicating attribution of outcomes solely to flavonoids. In contrast, Kasem et al. (2022) used pure hesperidin, providing more direct evidence of its effects. These differences emphasize the importance of compound purity and experimental design in interpreting hormonal results. Differences in extraction solvents as reported in some studies (aqueous, hydroethanolic, and methanolic) may alter the bioavailability and activity of the compounds, which in turn could hinder direct comparisons. Moreover, several studies used multi-component mixtures, such as *Ipomoea batatas* and *Murraya koenigii* (Kaur et al., 2020; Majid et al., 2019), complicating the identification of active ingredients.

## 5 Conclusion

Overall, the data suggest that flavonoids have the potential to mitigate BPA-induced reproductive toxicity in male rodents. Hormonal and oxidative stress pathways have been demonstrated to be the main targets of intervention. However, the variability in experimental protocols, compound purity, and organ-specific effects calls for more standardized and rigorous studies. The observed high heterogeneity across oxidative stress biomarkers reinforces the need for tissue-specific investigation and dose optimization in future studies. Additionally, understanding the biphasic and sometimes pro-oxidant nature of flavonoids is critical, particularly at high doses or in combination with other endocrine disruptors. Future research should prioritize longitudinal studies, dose–response assessments, and geographic diversification.

## Data Availability

The raw data supporting the conclusions of this article will be made available by the authors, without undue reservation.
